# Probing the antioxidant activity of functional proteins and bioactive peptides in *Hermetia illucens* larvae fed with food wastes

**DOI:** 10.1038/s41598-022-06668-9

**Published:** 2022-02-18

**Authors:** Jiaxin Lu, Yuwen Guo, Atif Muhmood, Bei Zeng, Yizhan Qiu, Pan Wang, Lianhai Ren

**Affiliations:** 1 School of Ecology and Environment, Beijing Technology and Business University, Beijing, 100048 China; 2 State Environmental Protection Key Laboratory of Food Chain Pollution Control, Beijing Technology and Business University, Beijing, 100048 China; 3 Key Laboratory of Cleaner Production and Integrated Resource Utilization of China National Light Industry, Beijing Technology and Business University, Beijing, 100048 China; 4 Institute of Soil Chemistry and Environmental Sciences, Ayub Agricultural Research Institute, Faisalabad, Pakistan

**Keywords:** Ecology, Environmental sciences

## Abstract

Food waste is becoming more prevalent, and managing it is one of the most important issues in terms of food safety. In this study, functional proteins and bioactive peptides produced from the enzymatic digestion of black soldier fly (*Hermetia illucens L.*, BSF) fed with food wastes were characterized and quantified using proteomics-based analysis. The results revealed approximately 78 peptides and 57 proteins, including 40S ribosomal protein S4, 60S ribosomal protein L8, ATP synthase subunit alpha, ribosomal protein S3, Histone H2A, NADP-glutamate dehydrogenase, Fumarate hydratase, RNA helicase, Chitin binding Peritrophin-A, Lectin C-type protein, etc. were found in BSF. Furthermore, functional analysis of the proteins revealed that the 60S ribosomal protein L5 (RpL5) in BSF interacted with a variety of ribosomal proteins and played a key role in the glycolytic process (AT14039p). Higher antioxidant activity was found in peptide sequences such as GYGFGGGAGCLSMDTGAHLNR, VVPSANRAMVGIVAGGGRIDKPILK, AGLQFPVGR, GFKDQIQDVFK, and GFKDQIQDVFK. It was concluded that the bioconversion of food wastes by BSF brought about the generation of a variety of functional proteins and bioactive peptides with strong antioxidant activity. However, more studies are required to exploit BSF's potential in the value addition of food wastes.

## Introduction

Food waste management is a major issue hampering the efficiency and sustainability of food supply chains. Improper food waste disposal can contribute to greenhouse gas (GHG) emissions, water depletion and threaten land use and biodiversity^1,2^. In most developed and developing countries, food waste is the most significant component of municipal solid waste^3^. Annually, around 100 million tons of food waste is generated in the European Union^4^. Currently, microbial technologies (e.g. anaerobic digestion, composting) treat food waste, converting organic matter into methane and fertilizer^5^. However, inadequate cellulose degradation, low purity of high-value products, and the tendency for fermentation systems to acidify are the major hindrances to the scaling up of these technologies^6^. Therefore, developing new technologies for treating and recycling food waste increases researchers' interest. Recently, transforming food waste into high-value products by feeding food waste to insects emerged as a beneficial strategy^7,8^. Moreover, insects' high food intake and low nutritional requirements help to dissipate food waste and convert it into high-value products such as oils and proteins^9–11^.

*Hermetia illucens* known as the Black Soldier Fly (BSF), is an insect belonging to the order of Dipterans and family of Stratiomyidae with a life cycle consisting of five stages; egg, larva, prepupa, pupa, and adult^12^. In treating solid waste, black soldier fly (*Hermetia illucens L*.) larvae (BSFL) offer benefits such as increased food waste reduction efficiency and reproductive capacity and reduced greenhouse gas emissions such as methane and carbon dioxide^13^. Studies have confirmed that treating pig manure with BSF reduces nitrogen by 55.2%, potassium by 52.8% and phosphorus by 44.3%^14^. Due to their simple feeding and high protein properties, insects were proposed by FAO/WHO in 2013 as sustainable protein substitutes^15^. BSF can be used as a feed additive for fish and poultry feeds because of its higher protein content (40–48%)^16^. In addition, BSF contained various bioactive peptides with antihypertensive, antimicrobial, antioxidant, anti-diabetic, immunomodulatory and mineral binding properties and could develop high-value products^17,18^.

Various functional components have been identified in insects, including fatty acids, flavonoids, polyphenols, proteins, antioxidant enzymes^19–21^. Enzymatic hydrolysis methods were found to have higher extraction efficiency^22^. Similarly, mass spectrometry is a powerful tool for identifying and quantifying immunoreactive proteins in a short time^23^. Likewise, it has a carefully curated database of all possible proteins present in an organism, a considerable advantage for peptide sequence identification. Some work has been conducted previously to identify bioactive peptides in different insects^24–27^; however, work on characterization and quantification of bioactive peptides in BSF feeding on food waste is relatively scarce.

Therefore, to address this knowledge gap, in this study, an integrated approach containing enzymatic hydrolysis and mass spectroscopy was employed for the efficient protein extraction and characterization of bioactive peptides from BSF feeding on food waste. Moreover, bioactive peptides functions, antioxidant properties, safety, and application prospects were also analyzed.

## Results and discussion

### Antioxidant activity of protein hydrolysate in BSF

In this study, the antioxidant properties of peptides in BSF extracts were determined by analyzing DPPH radical scavenging, ABTS radical scavenging and hydroxyl radical scavenging activities.

#### DPPH radical scavenging activity

**Figure 1:**
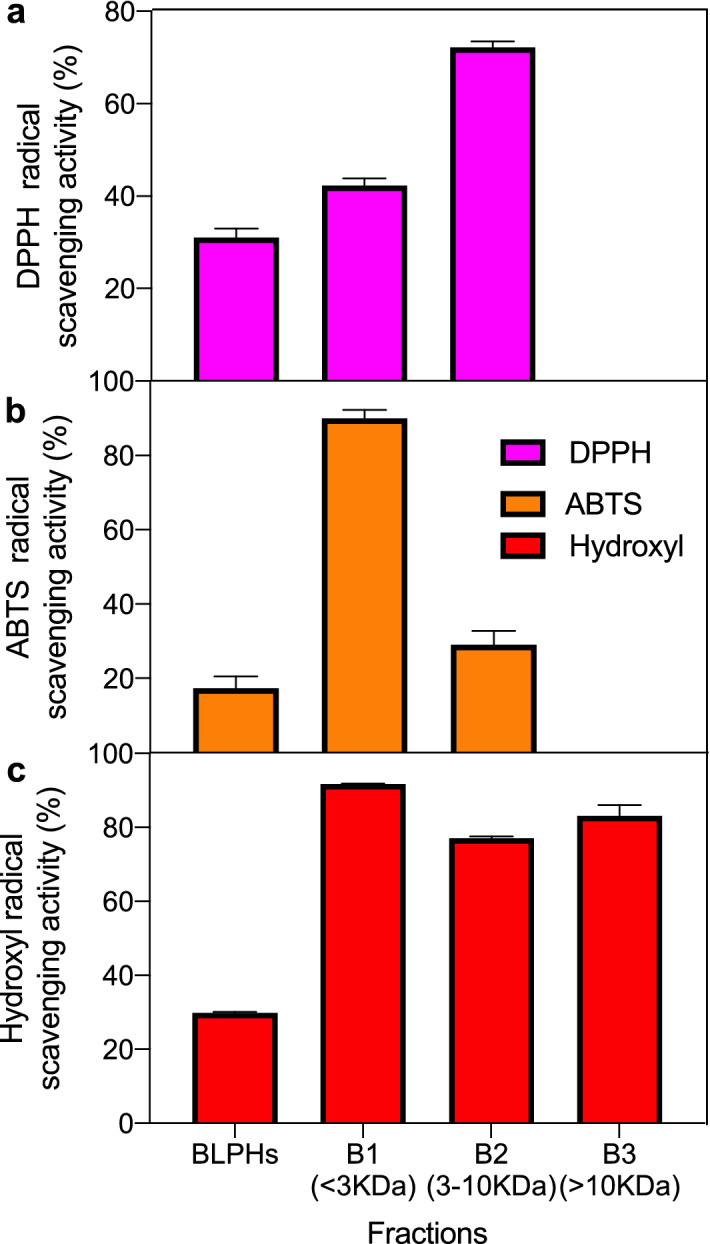
Free radical scavenging activities of BSF protein hydrolysates (BLPHs) and ultrafiltration of different fractions B1 (< 3 kDa), B2 (3–10 KDa), B3 (> 10 kDa). (**a**) DPPH radical scavenging activity; (**b**) ABTS radical scavenging activity; (**c**) hydroxyl radical scavenging activity (n = 3, *p* < 0.05, ANOVA).

DPPH radical scavenging activity is widely used to evaluate the antioxidant properties of natural extracts^28^. When encountering free radical scavengers, the lone pair of DPPH electrons are paired, making its absorbance at the maximum absorption wavelength smaller. The DPPH radical scavenging activity of BLPHs was 31.06%, while the radical scavenging activities of B-1 (< 3 kDa), B-2 (3–10 kDa) and B-3 (> 10 kDa) were 42.29%, 72.23% and 0, respectively (Fig. 1a). The DPPH radical scavenging ability of BSF was enhanced by ultrafiltration treatment. The low molecular weight B-1 and B-2 exhibited the highest DPPH radical scavenging properties. However, the protein hydrolysate of B-3 failed to remove the DPPH radical. Arise et al.^29^ reported that peptide size was inversely related to DPPH radical scavenging activity. Similar results, with a lower molecular weight peptide exhibiting a higher DPPH radical scavenging activity, have also been reported for protein hydrolysates of rapeseed^29^ and milk^30^. Liu et al.^31^ reported a higher DPPH scavenging activity of low molecular weight proteins (< < 3 kDa) from the extracts of male silkworm moths.

#### ABTS radical scavenging activity

ABTS radical scavenging activity can be applied to both lipophilic and hydrophilic compounds, and it is excellent in assessing the radical scavenging activity of protein hydrolysates and peptides^32^. As shown in Fig. 1b, the ABTS scavenging capacity of BLPHs was 17.34%, while the radical scavenging activities of B-1 (< 3 kDa), B-2 (3-10 kDa) and B-3 (> 10 kDa) were 89.99%, 29.00% and 0, respectively. B-1 showed the highest scavenging of ABTS, which may be due to smaller amino acids, including cysteine, tryptophan and tyrosine, amino acids with increased activity in removing ABTS^33,34^. Consistent with the results of the DPPH radical scavenging assay, protein hydrolysates of B-3 (MW > 10 kDa) failed to remove ABTS radicals.

#### Hydroxyl radicals scavenging activity

Hydroxyl radicals are the most reactive radicals and can react with most biomolecules, including peptides, proteins, lipids and DNA^35^. Hydroxyl radicals have the highest electron reduction potential^36^. Therefore, some antioxidants with strong electron transfer properties may be hydroxyl radical scavengers^37^. As shown in Fig. 1c, the hydroxyl radical scavenging capacity of BLPHs was 29.85%, and the radical scavenging activities of B-1 (< 3 kDa), B-2 (3–10 kDa) and B-3 (> 10 kDa) were 77.11%, 91.67% and 83.13%, respectively. The results were consistent with the free radical scavenging activities of DPPH and ABTS, with lower weight hydrolytic peptides showing better scavenging of hydroxyl radicals^38,39^.

### Identification and annotation of peptides in BSF extracts

**Figure 2:**
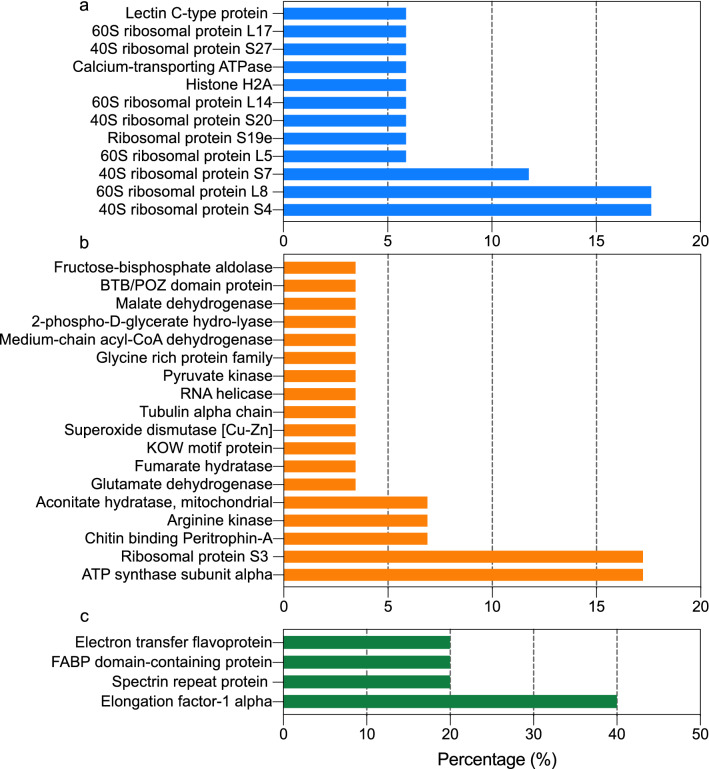
GO functional classification of BSF protein distribution based on the biological process (**a**), molecular function (**b**), and cellular localization (**c**).

Based on HPLC–ESI–MS/MS proteomics analysis, a total of 78 peptides were identified from the BSF extracts and classified into 57 proteins, as shown in Table S1. Gene ontology (GO) classification is commonly used to analyze the potential function of proteins^40^. The potential role of functional proteins in BSF was evaluated for GO functional classification based on their biological processes, cellular composition and molecular function. As shown in Fig. 2, the most significant clusters of biological processes include 40S ribosomal protein S4 (17.65%), 60S ribosomal protein L8 (17.65%) and 40S ribosomal protein S7 (11.77%) proteins, which are mainly composed of RNA and protein aggregation binding and contribute to the formation of large ribosomal subunits. Among the functional peptides, the most abundant clusters were ATP synthase subunit alpha (17.24%), Ribosomal protein S3 (17.24%), whose functions are to regulate ATP citrate lyase activity and contribute to the structural integrity of the ribosome, respectively^41,42^. The more abundant cluster in the cellular component was Elongation factor-1 alpha (40%), a eukaryotic translation elongation factor substance^43^.

### Proteomics functional analysis

**Figure 3:**
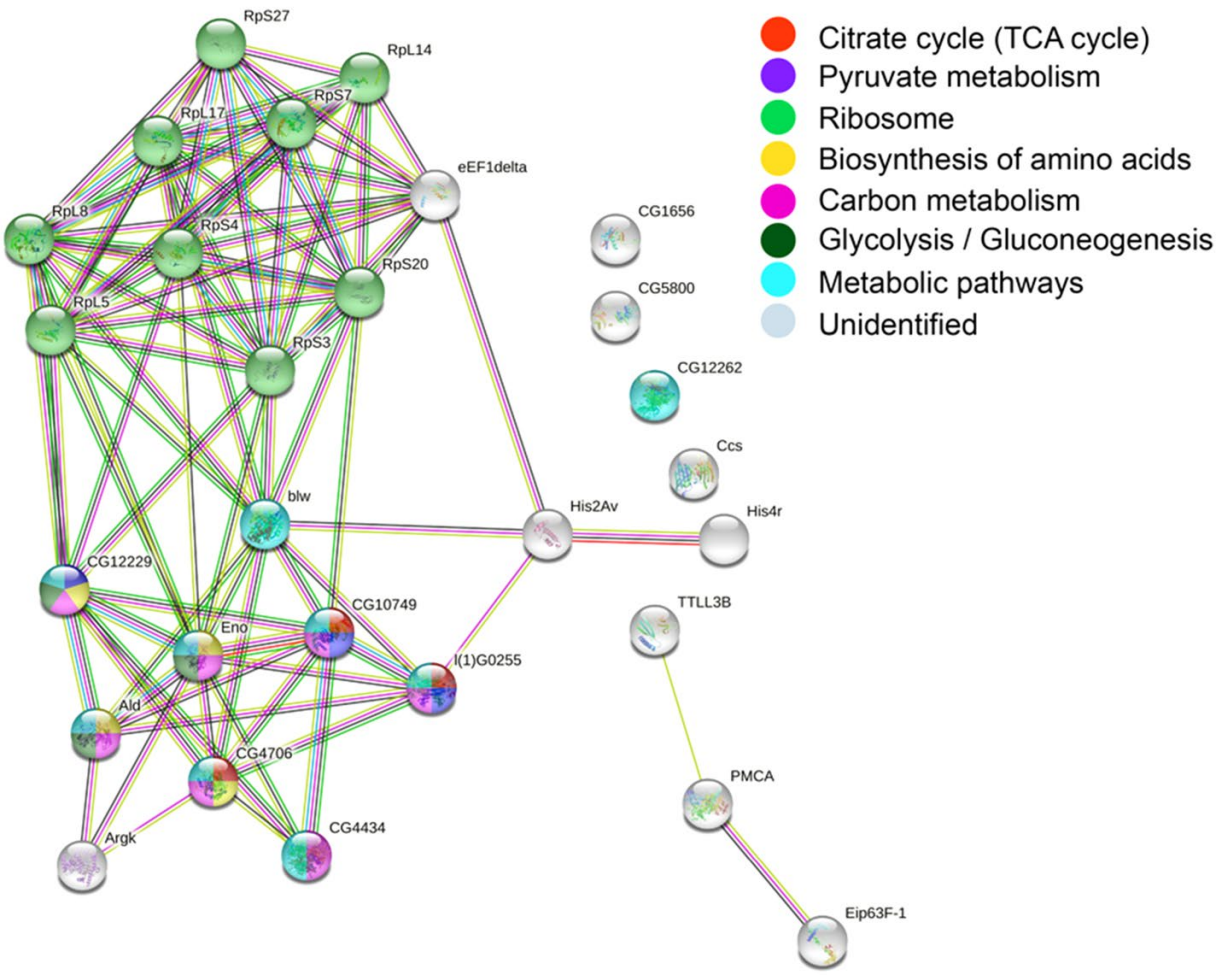
Protein interactome network for the BSF proteome using the STRING software. Stronger associations are represented by thicker lines.

To better understand the interaction of insect-derived proteins generated during the food waste treatment by BSF, we constructed a PPI network through the STRING following a previously reported method^44^. KEGG (Kyoto Encyclopedia of Genes and Genomes) pathway database (https://www.kegg.jp/kegg/kegg1.html) was used to perform metabolic pathway analysis. As shown in Fig. 3 and Table 1, these interactions were divided into seven KEGG metabolic pathways: Glycolysis/Gluconeogenesis, Citrate cycle (TCA cycle), Pyruvate metabolism, Metabolic pathways, Carbon metabolism, Biosynthesis of amino acids, Ribosome. Figure 3 shows a global view of the proteome and metabolic pathways in BSF. Each circle represents a phosphorylated protein, and the straight line represents the interaction between proteins; the more substantial the interaction between two proteins, the thicker the linkage. Pyruvate kinase (CG12229) was involved in Pyruvate metabolism, Carbon metabolism, Glycolysis/Gluconeogenesis, Biosynthesis of amino acids. 60S ribosomal protein L5 (RpL5) interacts with various ribosomal proteins and directs the glycolytic process of CG1229 (AT14039p), promoting potassium and magnesium ions and pyruvate kinase activity. It has been shown that 60S ribosomal protein L5 (RpL5) is a nucleoplasmic shuttle protein that plays a vital role in assembling large ribosomal subunits and in the intracellular transport of 5S rRNA^45^. Teng et al.^46^ found that deletion of RPL5 strongly inhibited cell cycle progression with effects on cell cycle progression arising from reduced ribosome content and translational capacity, which inhibited the accumulation of cell cycle proteins at the translational level.

**Table 1:** STRING-based KEGG metabolic pathway of the BSF proteome.

Pathway	Description	Count in network	Strength	False discovery rate
dme00010	Glycolysis/Gluconeogenesis	3 of 51	1.47	0.0127
dme00020	Citrate cycle (TCA cycle)	3 of 39	1.58	0.0089
dme00620	Pyruvate metabolism	3 of 43	1.54	0.0094
dme01100	Metabolic pathways	9 of 1102	0.61	0.0139
dme01200	Carbon metabolism	7 of 117	1.47	8.47E-07
dme01230	Biosynthesis of amino acids	4 of 65	1.49	0.0015
dme03010	Ribosome	9 of 132	1.53	2.25E-09

*BSF refers to Black soldier fly (*Hermetia illucens L*.).

The biological process involved in CG4434 (Glutamate dehydrogenase [NAD(P) +]) is described as a redox process, the catabolism of glutamate to 2-ketoglutarate, which interacts with CG10749 (L-malate dehydrogenase) to promote the life activities of the BSF. The two enzymatic reactions catalyzed by phosphofructokinase and glutamate dehydrogenase have activated glycolysis^47^. It is involved in the biological process described with: tricarboxylic acid cycle; malate metabolic process; cellular carbohydrate metabolic process.

### Antioxidant activity prediction analysis

The Peptide Ranker was used to score the peptide sequences identified by the mass spectrometry to predict the biological activity of BSF (Table 2). The amino acid sequence of the peptide was used to obtain a specific Peptide Ranker score between 0 and 1, with higher scores indicating a higher probability of being a biologically active peptide^48^. The Peptide Ranker prediction of BSF extracts identified 28 bioactive peptides with a probability higher than 0.30. The bioactive peptides were derived from 17 identified precursor proteins, including LIM domain protein, Elongation factor-1 alpha (Fragment), biological process protein (Histone H2A 60S ribosomal protein L8, 40S ribosomal protein S4, 60S ribosomal protein L14, Lectin C-type protein), molecular function proteins (Fumarate hydratase, RNA helicase, Ribosomal protein S3, Chitin-binding Peritrophic-A, Aconitate hydratase, mRNA helicase. Aconitate hydratase, mitochondrial, Arginine kinase).

**Table 2:** Predicted the bioactivity of the part differentially excreted peptides by peptide ranker (bioactivity > 0.50).

Peptide sequences	Predicted bioactivity	Protein name	Antioxidant sequences
GYGFGGGAGCLSMDTGAHLNR	0.83	LIM domain protein	HL, AH, GAH
AGLQFPVGR	0.76	Histone H2A	R,G
HFQAPSHIR	0.51	KOW motif protein	IR
VGIKAPGIIPR	0.48	ATP synthase subunit alpha	R,V
GFIGPGVDVPAPDMGTGER	0.47	Glutamate dehydrogenase (NAD(P)( +))	FIGP
SQINFPIGGPTER	0.46	Fumarate hydratase	R
AVDSLVPIGR	0.45	ATP synthase subunit alpha	R,V
VVPSANRAMVGIVAGGGRIDKPILK	0.44	60S ribosomal protein L8	LK, KP
GFKDQIQDVFK	0.41	RNA helicase	KD
TQLEPPISTPHCAR	0.37	Chitin binding Peritrophin-A	R
TIRYPDPLIK	0.36	40S ribosomal protein S4	IR, RY
SKIPFNVTPGSEQIR	0.35	Aconitate hydratase, mitochondrial	IR
RIPFSHDDR	0.32	Arginine kinase	R
VLVDGPLTGVPR	0.32	60S ribosomal protein L14	R
GVEEDWLSAR	0.31	Lectin C-type protei	G,R
IGGIGTVPVGR	0.30	Elongation factor-1 alpha	R,G

According to the BIOPEP database^49^, the antioxidant fragments in the BSF peptides were predicted and found to contain mainly glycine (G), proline (P), phenylalanine (F), arginine (R) basic amino acids, histidine (H), lysine (K) basic amino acids, tyrosine (Y), valine (V) and leucine (L). H, F, Y are aromatic amino acids. V and L are hydrophobic amino acids. G is an aliphatic amino acid. P is a heterocyclic amino acid. R and K are basic amino acids.

There have been numerous reports on the relationship between amino acids and antioxidant activity. The hydrophobic amino acids in peptides can enhance the antioxidant activity of peptides by increasing their solubility in lipids and by interacting with free radicals^50^. Overall, the peptides GYGFGGGAGCLSMDTGAHLNR and AGLQFPVGR were identified in this experiment with high activity scores, which may be attributed to the presence of the hydrophobic amino acids glycine (G) and arginine (R) at the N-terminal end of the peptide (Table 2). The single hydrogen atom of glycine (G) contributes to the flexibility of the peptide backbone and thus to the antioxidant properties. Arginine (R) has been reported to act as a free radical scavenger, inhibiting pro-oxidant active enzymes and indirectly acting as an antioxidant to scavenge oxygen-containing free radicals with high antioxidant activity. Lysine helps to remove reactive oxygen species (hydrogen oxides, superoxide and hydroxyl radicals.) in a Maillard reaction with other reducing sugars^51^. Thus VVPSANRAMVGIVAGGGRIDKPILK, GFKDQIQDVFK, GFKDQIQDVFK exhibit higher antioxidant properties because of the presence of K. Among the free amino acids tryptophan, Trp (W) is known to have the strongest uptake of oxygen radicals and is thought to be responsible for the high antioxidant activity of the peptide^52^.

### Safety evaluation of potentially active peptides

To further evaluate the safety of peptides with potential antioxidant activity extracted from BSF, an online software, ToxinPred and AllergenFP, were used to predict the peptides' allergenicity and toxicity (Table 3)^53^. The peptide sequences, including GYGFGGGAGCLSMDTGAHLNR, VVPSANRAMVGIVAGGGRIDKPILK, HFQAPSHIR, TIRYPDPLIK and IGGIGTVPVGR, with protein LIM domain protein, KOW motif protein, 60S ribosomal protein L8, 40S ribosomal protein S4, and Elongation factor-1 alpha (Fragment), were observed to have allergenic properties. These allergenic risk peptides need to be removed when developing active peptide products for BSF. In contrast, the antioxidant peptides AGLQFPVGR, VGIKAPGIIPR, GFIGPGVDVPAPDMGTGER, SQINFPIGGPTER, AVDSLVPIGR, GFKDQIQDVFK, TQLEPPISTPHCAR, SKIPFNVTPGSEQAR, SKIPFNVTPGSEQAR, SKIPFNVTPGSEQAR, SKIPFNVTPGSEQAR, SKIPFNVTPGSEQAR. SKIPFNVTPGSEQIR, VLVDGPLTGVPR, GVEEDWLSAR are non-toxic and non-allergenic. These peptides belong to Histone H2A, ATP synthase subunit alpha, NADP-glutamate dehydrogenase, Fumarate hydratase, RNA helicase, Chitin binding Peritrophin-A Aconitate hydratase, mitochondrial, 60S ribosomal protein L14, Lectin C-type protein.

**Table 3:** Safety evaluation of potential active peptides.

Peptide sequences	Allergenicity	Toxicity
GYGFGGGAGCLSMDTGAHLNR	Probable allergen	Non-Toxin
AGLQFPVGR	Probable non-allergen	Non-Toxin
HFQAPSHIR	Probable allergen	Non-Toxin
VGIKAPGIIPR	Probable non-allergen	Non-Toxin
GFIGPGVDVPAPDMGTGER	Probable non-allergen	Non-Toxin
SQINFPIGGPTER	Probable non-allergen	Non-Toxin
AVDSLVPIGR	Probable non-allergen	Non-Toxin
VVPSANRAMVGIVAGGGRIDKPILK	Probable allergen	Non-Toxin
GFKDQIQDVFK	Probable non-allergen	Non-Toxin
TQLEPPISTPHCAR	Probable non-allergen	Non-Toxin
TIRYPDPLIK	Probable allergen	Non-Toxin
SKIPFNVTPGSEQIR	Probable non-allergen	Non-Toxin
RIPFSHDDR	Probable non-allergen	Non-Toxin
VLVDGPLTGVPR	Probable non-allergen	Non-Toxin
GVEEDWLSAR	Probable non-allergen	Non-Toxin
IGGIGTVPVGR	Probable allergen	Non-Toxin

Histone H2A variants have also been reported to have anti-microbial activity in vertebrates and invertebrates by acting as antimicrobial peptides (AMP) in host immune responses^54^. Arockiaraj et al. identified Histone H2A with high immune activity in freshwater shrimp^55^. Fumarate hydratase is an enzyme of the tricarboxylic acid cycle (TCA cycle) that catalyzes the hydration of fumaric acid to malic acid^56^. Fumarate hydratase was detected as a major component of the antimicrobial peptide in juniper extracts^57^. RNA helicase can have a direct stimulatory effect on viruses^58^.

In contrast, chitin-binding Peritrophin-A is widely used in packaging, agriculture and cosmetics^59^. Aconitate hydratase Lectin C-type protein promotes wound healing and skin protection and has been detected in sea cucumbers^60,61^. Therefore, BSF feeding on food waste has high antioxidant activity and application potential in food processing, cosmetics, and pharmaceuticals.

## Conclusion

This study characterised the high-value antioxidant functional proteins and peptides of BSF based on HPLC–ESI–MS/MS proteomics analysis. Seventy-eight peptides and 57 proteins were identified from the BSF extracts. The most significant clusters of biological processes included 40S ribosomal protein S4 (17.65%), 60S ribosomal protein L8 (17.65%) and 40S ribosomal protein S7 (11.77%). Proteomic functional analysis revealed that the 60S ribosomal protein L5 (RpL5) in BSF could interact with the glycolytic process of CG1229 (AT14039p), thus facilitating the breakdown of glutamate to 2-ketoglutarate and other activities. Moreover, about 28 bioactive peptides with 17 precursor proteins were identified from peptide ranker prediction. Safety evaluation of bioactive peptides depicted some peptides with allergic risk, while most of the identified peptides were non-toxic and non-allergenic. Therefore, functional proteins and bioactive peptides in BSF feeding on food waste can be used in food processing, cosmetics, and medicine. Likewise, the use of food waste as feed for BSF is a promising value-addition strategy for better management of food wastes.

## Materials and methods

### Materials

Eggs of BSF were obtained from Changzhou Wiley Food Waste Treatment Plant (Jiangsu, China). Food waste was taken from the cafeteria of Beijing Industrial and Commercial University (Beijing, China) and used to culture BSF. Consecutive food waste collections were mixed, crushed and stored at − 20 °C. The incubation material consisted of wheat bran, peanut bran and water in a mass ratio of 1:1:13 with a moisture content of 67%.

### BSF sample preparation

#### Incubation of BSF

Freshly qualified eggs (the eggs having ability to be hatched) of BSF were obtained and incubated for three days at 30–32 °C. 0.10 g BSFL were carefully weighed using Mettler RT1200 electronic precision scales after incubation and placed in 150 g of incubation material at 30–32 °C for five days. To keep the material soft and wet, a little amount of water is sprayed over it. The BSF was collected after the BSFL had hatched for roughly seven days and had been raised on food waste for 14 days.

#### Preparation of BSF samples

To eliminate any residual food from the gastrointestinal system, the BSF were fasting for around 48 h. Freeze-drying was performed to eliminate water from the insects and keep the peptides functional in the BSF. For further investigation, approximately 100 g of BSF were freeze-dried, crushed, and kept at − 18 °C. Fats were extracted using the Folch technique [19], which involved combining the extractant (chloroform and methanol 4:1 v/v) with the BSFL powder in a 5:1 ratio, sonicating for 15 min at 100 w, centrifuging at 5000 r/min at 4 °C for 5 min and collecting the residue. The defatted BSF powder was dried at 50 °C to remove the extractant.

### Enzymatic extraction of BSF protein

#### Preparation of the enzymatic hydrolysate of BSF

At pH 8.0 and 55 °C, alkaline protease purchased from Beijing Biotopped Technology Co., LTD (Beijing, China) was used to hydrolyze the proteins. The enzyme/substrate ratio was 1.5:100 (w/v) for all hydrolysis processes, while the substrate/solvent ratio was 1:20 (w/v). The mixture was incubated for 2 h before heated for 10 min at 100 °C. The supernatant, which included BSFL protein hydrolysate solution (BLPHs), was freeze-dried and kept at − 20 °C after centrifugation for 10 min. The protein content of BLPHs was determined using the Bradford technique.

#### Ultrafiltration of hydrolysate

The hydrolysate was ultrafiltered using filters with a cut-off molecular weight of 3 kDa and 10 kDa, respectively, to obtain three ultrafiltration fractions, which were classified according to their molecular weight: < 3 kDa (B1), 3–10 kDa (B2) and > 10 kDa (B3)^62–64^.

### Determination of the antioxidant activity of BSF

#### Assay of 2,2′-diphenyl-1-picrylhydrazyl(DPPH)radical scavenging activity

The capacity of BLPHs to scavenge DPPH radicals was measured according to the method described by Chung et al.^65^. 1 mL of sample (initial protein content is 0.2 mg/mL) was mixed with 1 mL of DPPH-95% methanol solution, and the reaction was carried out for 30 min, then absorbance was measured at 515 nm. The cleaning effect was calculated using the formula:

$${\text{Scavenging }}\;{\text{activity}}\;{\text{ (\% )}} = \frac{{1{ } - {\text{A}}_{{{\text{sample}}}} }}{{{\text{A}}_{{{\text{control}}}} }} \times 100$$

A_sample_ is the absorbance of the DPPH solution with the sample; A_control_ is the absorbance of the DPPH solution without the sample.

#### 2,2′-azinobis (3-ethylbenzothiazoline 6-sulfonate) (ABTS) radical scavenging activity

The capacity of BLPHs to scavenge ABTS radicals was performed following the already reported procedure^66^. Briefly, equal volumes of ABTS solution and K_2_S_2_O_8_ were reacted in the dark for 12 h at 25 °C, then diluted with phosphate buffer (PBS) until their absorbance at 734 nm reached 0.7 ± 0.02. Then 0.2 mL of sample (1 mg⋅mL^−1^) was added to 0.8 mL of working solution held at 25 °C for 5 min. The absorbance was measured at 734 nm to calculate the total antioxidant capacity.

$${\text{Scavenging}}\;{\text{ activity }}\;{\text{(\% ) }} = \frac{{{\text{A}}_{{{\text{control}}}} - {\text{A}}_{{{\text{sample}}}} }}{{{\text{A}}_{{{\text{control}}}} }} \times 100$$

A_sample_ is the absorbance of the ABTs solution with the sample; A_control_ is the absorbance of the ABTs solution with 95% ethanol.

#### Hydroxyl radicals scavenging activity

The capacity of BLPHs to scavenge hydroxyl radicals was evaluated according to the previous method^67^. The samples, FeSO_4_ and salicylic acid–ethanol solution were mixed at a ratio of 1:1:1. Then 1.0 mL of H_2_O_2_ was added and incubated in a water bath at 37 °C for 30 min. Absorbance was measured at 510 nm using a spectrophotometer. Ascorbic acid was used as a positive control. The hydroxyl radical scavenging activity was calculated using the formula

$${\text{Scavenging}}\;{\text{ activity }}\;{\text{(\% ) }} = \frac{{{\text{A}}_{{{\text{2}}}} - {\text{A}}_{{{\text{1}}}} }} {{{\text{A}}_{{{\text{1}}}} }} \times 100$$

A_2_ is the absorbance of the sample; A_1_ is the absorbance of the solution without the sample.

### Peptide identification by LC–MS/MS

The UHPLC-QTOF-MS/MS (1290 Infinity Series, Agilent Technologies, Santa Clara, CA, USA) was used to determine proteins in BSF extracts. The chromatograph consisted of a binary pump, a thermostat and an autosampler coupled with a 6550 UHD iFunnel Q-TOF liquid mass spectrometry system. Compounds were ionized by electrospray ionization (ESI) using a Jet Stream Technology ion source. Chromatographic separation was performed on a 2.1 × 150 mm, 1.8 µm particle size Agilent RRHD Eclipse Plus C18 column. Instrument control and data acquisition were performed using Agilent Mass Hunter workstation software. LC parameters were set as follows: 10 µL injection volume, 0.3 mL⋅minˉ^1^ mobile phase flow. The mobile phase consisted of 0.1% formic acid aqueous solution (solvent A) and 0.1% formic acid acetonitrile solution (solvent B). The gradient was applied as follows: 0–4 min at 3% B; 4–42 min at 35% B; 42–47 min at 40% B; 47–52 min at 90% B; 52–55 min at 90% B and run at 3% B Run for 5 min. The ion source gas (nitrogen) was set to 250 °C at a flow rate of 14 L min^−1^, the nebulizer pressure was 37 psi, the sheath gas temperature was 250 °C, and the sheath gas flow rate was 11 L min^−1^. The capillary voltage was set to 3500 V while the nozzle voltage was set to 1000 V and the voltage of fragmentor to 400 V. Positive ions were collected in MS scan mode and automated MS/MS mode at a scan rate of 9 scan/s for MS and 7 scan/s for MS/MS. Internal mass calibration was enabled, using two reference ions at m/z 121.0509 and 922.0098.

UniProt KB/Swiss-Prot database searches were performed for protein and peptide identification using the Spectrum Mill MS Proteomics Workbench (Agilent Technologies) with a 50 ppm mass tolerance. MS/MS search results were validated using the Spectrum Mill automated threshold strategy and peptide model to optimize fractions and R1 automatically-species and protein specificity of selected peptides in FASTA format were searched against the NCBInr database using the Protein BLAST search tool and the Blastp algorithm (US National Library of Medicine).

### Proteomics functional and Antioxidant activity prediction analysis

To better understand the interaction of insect-derived proteins generated during the food waste treatment by BSF, we constructed a PPI network through the STRING following a previously reported method^44^. While the Peptide Ranker was used to score the peptide sequences identified by the mass spectrometry to predict the biological activity of BSF.

### Safety evaluation of potentially active peptides

To further evaluate the safety of peptides with potential antioxidant activity extracted from BSF, an online software, ToxinPred and AllergenFP were used to predict the peptides' allergenicity and toxicity.

### Statistical analysis

One way analysis of variance (ANOVA) was used to evaluate significant differences between various parameters. The significant differences in all comparisons were set at p < 0.05. GraphPad Prism 8 was used for plotting and data analyses.

## Supplementary Information

Supplementary Information.
