# Supplementary material for: Probing the antioxidant activity of functional proteins and bioactive peptides in Hermetia illucens larvae fed with food wastes
Source: Sci Rep. 2022 Feb 18;12:2799. doi: 10.1038/s41598-022-06668-9 (PMC8857240; doi:10.1038/s41598-022-06668-9)
Supplement: Supplementary file 1 — Supplementary Information. [file 41598_2022_6668_MOESM1_ESM.docx]

**Table S1 Peptide sequences identified in BSF (p<0.05)**

| **Accessions** | **Sequence** | **PSMs** | **Proteins** | **Area** | **PEP** | **XCorr** | **Charge** | **MH+ [Da]** | **ΔM [ppm]** | **RT [min]** | **Missed Cleavages** |
| --- | --- | --- | --- | --- | --- | --- | --- | --- | --- | --- | --- |
| A0A1I8M7W7 | AVFPSIVGRPR | 4 | 7 | 9.232E+09 | 0.004693 | 3.29 | 2 | 1198.69475 | -9.03 | 69.87 | 0 |
| A0A1I8M7W7 | SYELPDGQVITIGNER | 5 | 7 | 4.775E+08 | 0.0006893 | 2.46 | 2 | 1790.87468 | -9.67 | 102.56 | 0 |
| A0A1I8M7W7 | AGFAGDDAPR | 1 | 7 | 4.302E+06 | 0.004338 | 2.28 | 2 | 976.43883 | -9.75 | 39.31 | 0 |
| A0A1I8M7W7 | VAPEEHPVLLTEAPLNPK | 1 | 7 | 8.671E+07 | 0.0271 | 2.20 | 3 | 1954.04605 | -9.44 | 93.87 | 0 |
| A0A1I8M7W7 | DSYVGDEAQSKR | 1 | 5 | 3.474E+06 | 0.1515 | 2.14 | 2 | 1354.61247 | -8.07 | 30.33 | 1 |
| A0A1I8M401 | NKDPLNDTVVDQFK | 1 | 30 | 7.280E+07 | 0.0004791 | 2.96 | 2 | 1632.80791 | -9.15 | 85.18 | 1 |
| A0A1I8M401 | AFDKIIGEWK | 2 | 30 | 3.408E+08 | 0.03683 | 2.79 | 2 | 1206.64043 | -9.43 | 91.31 | 1 |
| A0A1I8M401 | NKDPLNDTVVDQFKK | 1 | 30 | 1.071E+08 | 0.001055 | 2.47 | 2 | 1760.90178 | -9.11 | 69.60 | 2 |
| A0A1I8M401 | MQDLVDKLQQK | 1 | 30 | 1.945E+08 | 0.06032 | 2.44 | 2 | 1345.70268 | -8.77 | 63.42 | 1 |
| A0A1I8M401 | DLEEANIQHESTLANLR | 1 | 30 | 3.578E+07 | 0.01844 | 2.10 | 3 | 1952.94870 | -9.52 | 93.05 | 0 |
| T1PGB4 | TGAIVDVPVGDELLGR | 1 | 1 | 1.566E+08 | 0.002401 | 2.99 | 2 | 1610.86113 | -8.55 | 104.34 | 0 |
| T1PGB4 | AVDSLVPIGR | 2 | 1 | 1.814E+09 | 0.003728 | 2.79 | 2 | 1026.58440 | -9.63 | 85.34 | 0 |
| T1PGB4 | VGIKAPGIIPR | 2 | 1 | 7.468E+08 | 0.003182 | 2.72 | 3 | 1120.71018 | -8.91 | 69.82 | 1 |
| T1P7I8 | IGPKKPLPDNVSVVEPK | 3 | 2 | 2.833E+08 | 0.0001572 | 3.13 | 3 | 1817.03720 | -8.81 | 67.48 | 1 |
| T1P7I8 | KPLPDNVSVVEPK | 2 | 2 | 3.833E+08 | 0.1143 | 2.40 | 2 | 1421.78703 | -9.07 | 64.00 | 0 |
| A0A1I8MAP3 | LKDVGTEHEAIVVHGSYSWVDEK | 1 | 2 | 1.850E+07 | 0.004157 | 3.51 | 3 | 2598.25687 | -10.27 | 83.59 | 1 |
| A0A1I8MAP3 | DVGTEHEAIVVHGSYSWVDEK | 2 | 2 | 3.451E+07 | 0.004731 | 3.39 | 2 | 2357.08257 | -9.31 | 92.84 | 0 |
| A0A1I8NFN4 | VEVPVHVPVDRPYPVK | 1 | 3 | 5.800E+07 | 0.004591 | 2.87 | 4 | 1830.00905 | -9.98 | 86.45 | 0 |
| A0A1I8NFN4 | KVEVPVHVPVDRPYPVK | 1 | 3 | 1.132E+08 | 0.001086 | 2.61 | 4 | 1958.10341 | -9.64 | 75.60 | 1 |
| A0A1I8NFN4 | VDRPYPVPVDRPYPVK | 1 | 3 | 2.601E+08 | 0.04219 | 2.35 | 3 | 1897.01676 | -8.63 | 73.37 | 0 |
| T1PFA2 | TIRYPDPLIK | 3 | 2 | 1.958E+08 | 0.002061 | 2.80 | 3 | 1215.69797 | -9.59 | 72.15 | 1 |
| T1PAR6 | GVVKEIIHDPGR | 2 | 1 | 5.333E+06 | 0.01205 | 2.90 | 2 | 1319.73210 | -8.32 | 52.87 | 1 |
| T1PAR6 | VVPSANRAMVGIVAGGGRIDKPILK | 1 | 1 | 1.083E+08 | 0.1032 | 2.06 | 3 | 2518.42582 | -15.63 | 97.82 | 2 |
| A0A1I8MRC9 | IVELEEELR | 2 | 23 | 1.288E+08 | 0.03022 | 2.33 | 2 | 1129.59966 | -9.15 | 86.19 | 0 |
| A0A1I8MRC9 | KLAmVEADLER | 1 | 16 | 6.057E+06 | 0.01408 | 2.24 | 2 | 1290.66106 | -8.71 | 55.21 | 1 |
| T1PMI6 | TQLEPPISTPHcAR | 2 | 2 | 9.819E+07 | 0.00173 | 3.12 | 2 | 1606.78496 | -9.77 | 61.08 | 0 |
| A0A1I8MYL6 | HIDEMPFGYPFDR | 1 | 2 | 1.854E+08 | 0.000014739 | 3.39 | 2 | 1623.70964 | -10.14 | 102.55 | 0 |
| A0A1I8MYL6 | HIDEmPFGYPFDR | 1 | 2 | 3.545E+08 | 0.005394 | 2.56 | 2 | 1639.70561 | -9.40 | 99.17 | 0 |
| Q0Z9K0 | IGGIGTVPVGR | 1 | 5 | 3.214E+09 | 0.00003627 | 3.48 | 2 | 1025.60124 | -8.80 | 70.94 | 0 |
| Q0Z9K0 | LPLQDVYK | 1 | 5 | 1.259E+09 | 0.1482 | 1.83 | 2 | 975.54204 | -9.18 | 72.63 | 0 |
| T1PHS3 | TFDNDGLIDGDKFR | 3 | 1 | 2.945E+08 | 0.02041 | 2.94 | 2 | 1612.74639 | -8.60 | 92.80 | 1 |
| A0A1I8MCI4 | VIFIKGPENR | 2 | 2 | 2.384E+09 | 0.03675 | 2.59 | 2 | 1172.66753 | -9.51 | 56.13 | 1 |
| A0A1I8MUA4 | FNGVHIPGSPFR | 2 | 10 | 9.142E+07 | 0.01706 | 2.52 | 3 | 1327.67905 | -8.73 | 93.09 | 0 |
| A0A1I8MUA4 | FADHHVEGSPFTVK | 1 | 10 | 2.912E+07 | 0.0004263 | 2.46 | 3 | 1570.74424 | -13.18 | 57.76 | 0 |
| L0ESP4 | VSSTLSGLEGELK | 1 | 2 | 1.662E+08 | 0.0007544 | 2.55 | 2 | 1319.69316 | -9.24 | 92.87 | 0 |
| L0ESP4 | RIPFSHDDR | 1 | 2 | 1.116E+06 | 0.1594 | 2.39 | 3 | 1142.56006 | -8.86 | 33.00 | 1 |
| A0A1I8N262 | IPFNVTPGSEQIR | 1 | 2 | 6.710E+07 | 0.163 | 2.55 | 2 | 1457.76311 | -8.00 | 94.13 | 0 |
| A0A1I8N262 | SKIPFNVTPGSEQIR | 1 | 2 | 8.769E+07 | 0.05444 | 2.36 | 3 | 1672.88651 | -9.12 | 81.53 | 1 |
| T1PH01 | IIKPGGAEPDDFEK | 2 | 1 | 4.607E+07 | 0.00636 | 2.45 | 2 | 1515.75530 | -9.07 | 60.09 | 0 |
| A0A1I8MGJ3 | AVVAPVVTPVVK | 2 | 2 | 2.257E+08 | 0.04109 | 2.24 | 2 | 1178.74053 | -8.71 | 84.88 | 0 |
| T1PBD9 | GFIGPGVDVPAPDmGTGER | 1 | 2 | 5.418E+07 | 0.0003751 | 3.81 | 2 | 1887.87236 | -9.70 | 101.23 | 0 |
| T1PF40 | GAVDGGLNIPHSVK | 1 | 1 | 1.718E+08 | 0.00685 | 3.78 | 2 | 1363.72002 | -9.45 | 66.30 | 0 |
| T1PF92 | LKVPDQMDIIK | 1 | 1 | 9.669E+06 | 0.01258 | 3.33 | 3 | 1299.72242 | -9.04 | 90.36 | 1 |
| A0A1I8MGA2 | SQINFPIGGPTER | 1 | 2 | 3.028E+08 | 0.001431 | 3.11 | 2 | 1415.71538 | -8.79 | 92.97 | 0 |
| A0A1I8NCL8 | ENLDIDFDPDDK | 1 | 1 | 1.157E+07 | 0.08579 | 2.90 | 2 | 1435.61077 | -8.12 | 103.76 | 0 |
| T1PLF1 | GYGFGGGAGcLSmDTGAHLNR | 1 | 2 | 6.733E+06 | 0.00002105 | 2.84 | 2 | 2113.90312 | -7.01 | 82.03 | 0 |
| T1PEU3 | mLDPDDLINTPKPDER | 1 | 2 | 8.688E+07 | 0.007175 | 2.77 | 2 | 1884.88127 | -10.40 | 95.59 | 0 |
| T1PEZ2 | IIDLHSPSEIVKK | 1 | 1 | 2.702E+07 | 0.0252 | 2.75 | 2 | 1478.84282 | -10.11 | 66.17 | 1 |
| T1PHJ1 | VLVDGPLTGVPR | 1 | 1 | 1.540E+08 | 0.001212 | 2.75 | 2 | 1222.70427 | -9.16 | 97.41 | 0 |
| T1P7S8 | HFQAPSHIR | 1 | 1 | 1.102E+06 | 0.05608 | 2.70 | 3 | 1092.56055 | -8.44 | 32.00 | 0 |
| A0A1I8M153 | NVGTEEEAIVVK | 1 | 1 | 0.000E+00 | 0.002008 | 2.68 | 2 | 1287.66692 | -9.49 | 66.07 | 0 |
| A0A1I8N2Y3 | DNIQGITKPAIR | 1 | 5 | 9.568E+07 | 0.04208 | 2.60 | 2 | 1325.74138 | -9.25 | 60.58 | 0 |
| A0A1I8MA39 | TVVVHADPDDLGKGGHELSK | 1 | 3 | 2.211E+07 | 0.0001344 | 2.60 | 3 | 2074.03598 | -9.87 | 57.87 | 1 |
| A0A1I8NL20 | AGLQFPVGR | 1 | 4 | 1.998E+09 | 0.03889 | 2.54 | 2 | 944.52294 | -8.84 | 76.28 | 0 |
| T1PH24 | AVFVDLEPTVVDEVR | 1 | 1 | 1.791E+07 | 0.005052 | 2.53 | 2 | 1687.87370 | -9.79 | 104.46 | 0 |
| T1P8Z8 | GFKDQIQDVFK | 1 | 1 | 7.175E+07 | 0.1018 | 2.50 | 2 | 1324.67815 | -8.68 | 94.72 | 1 |
| A0A1I8NEK3 | NVAVITLNRPK | 1 | 2 | 6.375E+06 | 0.005388 | 2.47 | 2 | 1224.73076 | -9.46 | 57.27 | 0 |
| A0A1I8MEG4 | GVNLPGVPVDLPAVSEK | 1 | 3 | 1.798E+07 | 0.084 | 2.45 | 2 | 1690.91997 | -10.37 | 104.07 | 0 |
| T1PDF1 | DLEAIVQPIIAK | 1 | 2 | 3.564E+07 | 0.0776 | 2.43 | 2 | 1309.75994 | -9.71 | 104.87 | 0 |
| T1PH83 | VPVHVPQPYPVEK | 1 | 2 | 1.238E+08 | 0.00122 | 2.42 | 3 | 1488.80738 | -9.14 | 71.24 | 0 |
| A0A1I8MER1 | GVVIGTGLNTAIGK | 1 | 2 | 4.011E+07 | 0.006868 | 2.37 | 2 | 1299.75151 | -8.95 | 92.76 | 0 |
| A0A1I8N9I7 | DLLHPLPAQEK | 1 | 1 | 1.401E+08 | 0.1105 | 2.35 | 2 | 1260.68279 | -9.47 | 72.97 | 0 |
| T1PAW1 | GITFEDVRVPK | 1 | 2 | 4.853E+07 | 0.01291 | 2.33 | 2 | 1260.68254 | -9.67 | 80.29 | 1 |
| A0A1I8N4M6 | WYDNVITVGR | 1 | 2 | 4.624E+07 | 0.02135 | 2.31 | 2 | 1222.61089 | -8.71 | 98.11 | 0 |
| A0A1I8M6S3 | LGEEFDEETLDGR | 1 | 2 | 1.432E+07 | 0.01132 | 2.23 | 2 | 1509.65947 | -7.27 | 93.43 | 0 |
| A0A1I8MYN5 | LFEGGWEELGKEVNEK | 1 | 7 | 9.574E+06 | 0.00003015 | 2.19 | 2 | 1863.88664 | -13.83 | 101.38 | 1 |
| T1PF74 | LVVDHIQVNR | 1 | 1 | 1.315E+07 | 0.07973 | 2.11 | 2 | 1192.66765 | -10.13 | 60.32 | 0 |
| A0A1I8MSB3 | YRPLPPPPRPPR | 1 | 2 | 2.325E+07 | 0.04916 | 2.05 | 3 | 1442.82611 | -8.22 | 51.97 | 0 |
| A0A1I8N2V1 | IVDYTSDPVNGFNAVVR | 1 | 5 | 1.761E+07 | 0.00461 | 1.98 | 2 | 1865.91387 | -13.62 | 102.52 | 0 |
| T1PF13 | DIILPVPAFNVINGGSHAGNK | 1 | 2 | 1.858E+07 | 0.005943 | 1.98 | 2 | 2133.12407 | -9.92 | 104.94 | 0 |
| A0A1I8NGI3 | EVVPTTDPAVAFK | 1 | 2 | 4.359E+07 | 0.02367 | 1.70 | 2 | 1373.71892 | -8.93 | 95.53 | 0 |
| T1PHZ9 | EYDLLQPIHVSK | 1 | 1 | 7.312E+06 | 0.04653 | 1.67 | 2 | 1441.75127 | -12.02 | 96.12 | 0 |
| T1PIW2 | GVEEDWLSAR | 1 | 1 | 2.040E+07 | 0.1566 | 1.61 | 2 | 1161.54485 | -7.48 | 88.97 | 0 |
| A0A1I8MHN8 | NFLSRGHLTPDADFMFGYEQLSTYYYmNVAPQFQPINGGNWLKVEDMAR | 1 | 1 | 7.856E+06 | 0.1226 | 2.16 | 9 | 5747.77302 | 15.78 | 46.61 | 2 |
| T1PDM3 | LmQSSmELIAGHTR | 1 | 1 | 0.000E+00 | 0.1499 | 1.42 | 2 | 1605.74126 | -19.41 | 98.12 | 0 |
| A0A1I8MI51 | RGIIPGIKVDK | 1 | 3 | 1.107E+07 | 0.02957 | 1.85 | 3 | 1195.74103 | -9.33 | 55.31 | 2 |
| T1PBE8 | EVHAKDALDVYIEHRLLMESR | 1 | 1 | 0.000E+00 | 0.05863 | 0.46 | 2 | 2524.34746 | 19.68 | 102.44 | 2 |
| T1PEC8 | VVKGPVKPAKPPVFTK | 1 | 4 | 1.719E+06 | 0.04631 | 2.19 | 4 | 1692.04275 | -8.53 | 47.83 | 1 |

* PSMs is the number of identified secondary proteins belonging to the protein; Area is the peak area; PEP is the probability that a peptide (PSM-peptide spectral match) is incorrect; XCorr is the software score of the peptide, representing the confidence level of the peptide; Charge is the peptide MH+ [Da] is the molecular weight of the peptide; ΔM [ppm] is the measurement error of the molecular weight of the peptide; RT [min] is the retention time of the peptide on liquid chromatography; Missed Cleavages is the number of missed cleavage sites.
